# Supplementary material for: A metasurface-based full-color circular auto-focusing Airy beam transmitter for stable high-speed underwater wireless optical communications
Source: Nat Commun. 2024 Apr 5;15:2944. doi: 10.1038/s41467-024-47105-x (PMC10997589; doi:10.1038/s41467-024-47105-x)
Supplement: Supplementary file 1 — Supplementary Information [file 41467_2024_47105_MOESM1_ESM.docx]

**Supplementary Information for**

**A metasurface-based full-color circular auto-focusing Airy beams transmitter for stable high-speed underwater wireless optical communication**

Junhui Hu^1,#^, Zeyuan Guo^2,#^, Jianyang Shi^1^, Xiong Jiang^2^, Qinmiao Chen^2^, Hui Chen^3^, Zhixue He^3^, Qinghai Song^2,3^, Shumin Xiao^2,3,*^, Shaohua Yu^1,3^, Nan Chi^1,3,*^ and Chao Shen^1,3,*^

^1^Key Laboratory for Information Science of Electromagnetic Waves (MoE), School of Information Science and Technology, Fudan University, Shanghai, China

^2^Ministry of Industry and Information Technology Key Lab of Micro-Nano Optoelectronic Information System, Guangdong Provincial Key Laboratory of Semiconductor Optoelectronic Materials and Intelligent Photonic Systems, Harbin Institute of Technology, Shenzhen, China.

^3^Peng Cheng Laboratory, Shenzhen, China

#These authors contributed equally to this work.

**Corresponding authors*: shumin.xiao@hit.edu.cn; nanchi@fudan.edu.cn; chaoshen@fudan.edu.cn

**1. Metasurface designed for phase distribution of circular auto-focusing Airy beam**

The mathematical method of generating circular auto-focusing Airy beam (CAFAB) using metasurfaces is introduced. For the paraxial diffraction equation in one-dimensional space, the non-dispersive solution of the Airy function *h* (*s*, *ξ*) can be expressed as:

$h(s,\xi)=Ai\left[s-\left(\frac{\xi}{2}\right)^{2}\right]\exp\left(is\frac{\xi}{2}-i\frac{\xi^{3}}{12}\right)$ (S1)

where, *Ai*(.) is the Airy function, *s*=*x*/*x*_0_ is the one-dimensional dimensionless horizontal coordinate, *ξ* is the propagation distance. *x*_0_ is the normalized horizontal scale. However, this Airy function has infinite energy and cannot be realized in experiments. In general, its energy can be limited by introducing an exponential function:

$h(s,\xi=0)=Ai(s)\exp(as)$ (S2)

where *a* is the attenuation factor and satisfies *a*<<1 and *a*>0. The Fourier transform (FT) of the Airy wave packet at the origin *H*(*k*) is:

$H(k)=b\exp\left[\frac{i(2\pi b)^{3}k^{3}}{3}\right]$ (S3)

where *b* is a constant, and the unit is m^-1^. Eqs. (S3) shows that the FT of the Airy function has a cubic phase. In order to generate a circular Airy beam, we combine the cubic phase with the phase of the Fresnel lens and introduce it into the polar coordinate system. As shown in Fig. 2a, it can be expressed mathematically as:

 (S4)

where *f* is the focal length of the Fresnel lens, *r* is the coordinate with the center of the metasurface as the origin, *r*_0_ is the main lobe radius of the Airy beam, and *λ* is the wavelength of the incident light.

**2. Measurement of RGB lasers performance versus different water quality**


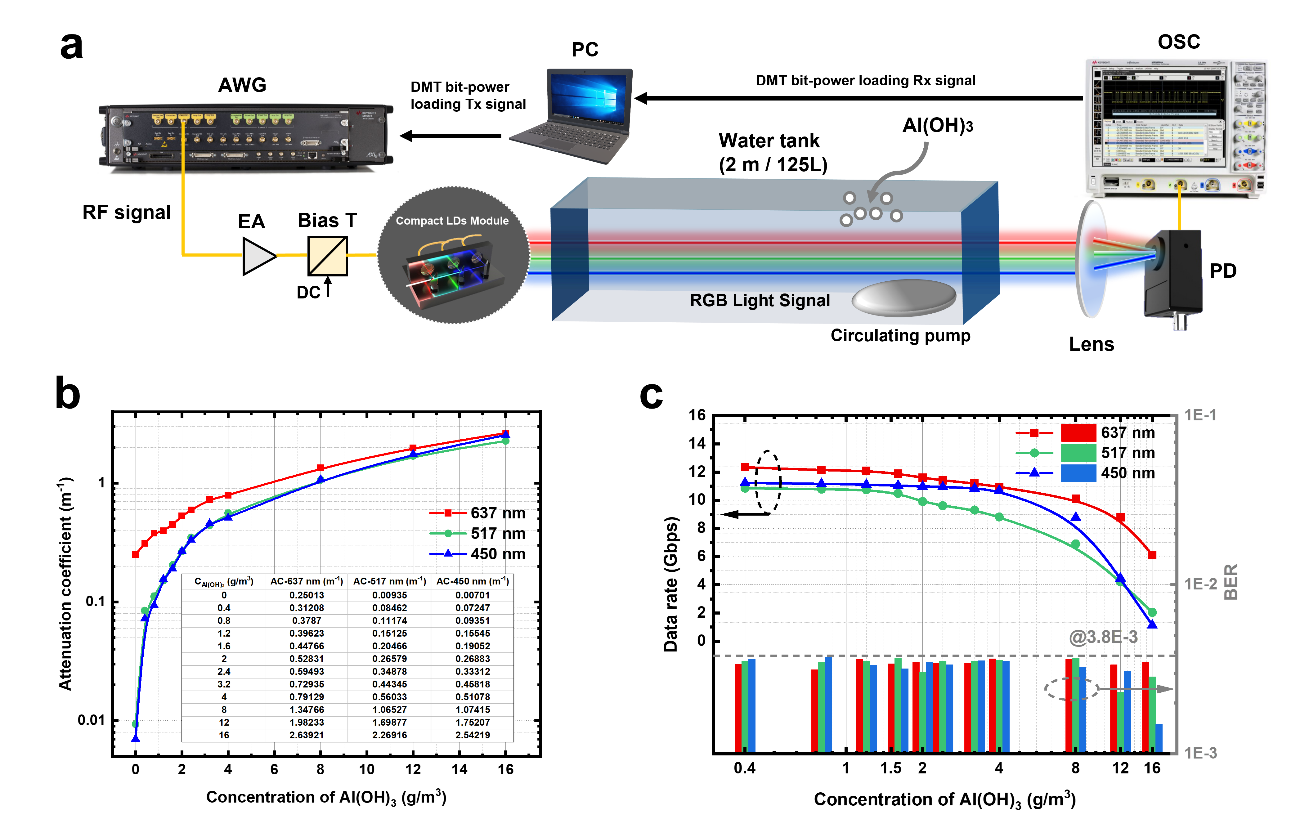


**Fig. S1. The effect of the concentration of different impurities on the attenuation coefficient and data rate of RGB beam.** **a** Experimental setup. **b** attenuation coefficient versus concentration of Al(OH)_3_. **c** Data rate versus concentration of Al(OH)_3_.

The Experimental platform is established as shown in Fig. S1(a). Firstly, import the coded bit-power loading discrete multitone (DMT) modulation format signal into the arbitrary waveform generator (AWG) on the PC side. After digital-to-analog (D/A) conversion, the modulated RF signal is amplified by the EA module and added to the RF port of the three-port BiasT, and added to the laser bias by inputting DC current. Then, the RF+DC signal is directly modulated on the laser through the SMA port. The RGB light signals pass through a 2-m water tank to simulate the underwater communication environment. In order to achieve different turbid water quality effects, we add different masses of Al(OH)_3_ particles to the water to achieve different suspension concentrations: 0 (pure water), 0.4, 0.8, 1.2, 1.6, 2, 2.4, 3.2, 4, 8, 12, 16 g/m^3^. A circulating pump is placed at the bottom of the water tank to evenly stir the particles. Throughout a lens at the receiving end, the RGB light signals are respectively received by a photodetector (PD). The electrical signal after photoelectric conversion is displayed on a digital storage oscilloscope (OSC). The PC side collects the Rx signal on the OSC, synchronizes it with the Tx signal, then calculates the bit error rate (BER) after demodulation and equalization. Fig. S1(b) depicts the attenuation coefficients of RGB beams as a function of Al(OH)_3_ concentration. The findings reveal notable advantages in the attenuation coefficients of blue-green beams (0.007, 0.009) compared to the red beam (0.25) in pure water. However, when the impurity concentration surpasses 8 g/m^3^, the attenuation coefficient of the blue beam exceeds that of the green beam and gradually approaches the red beam's value. These outcomes indicate that the attenuation window gradually shifts towards longer wavelengths as the water quality becomes more turbid. These experimental results align with previous work**^错误!未找到引用源。^**. Fig. S1(c) shows the transmission data rates of RGB lasers corresponding to different water qualities. The results demonstrate that in water environments with impurity concentrations exceeding 4 g/m^3^, the attenuation rate of blue-green beams surpasses that of red beam. This discrepancy arises because the RGB spectral attenuation coefficients are close, while the short distance further reduces their differences, and mature Si-based detectors exhibit greater sensitivity to longer wavelengths. Therefore, Therefore, red beam is more suitable for high-speed communication in short-distance turbid waters, and blue-green beams have an advantage in attenuation length in long-distance situations.

**3. Simulation of CAFAB and Gaussian beam obstacle avoidance characteristics**


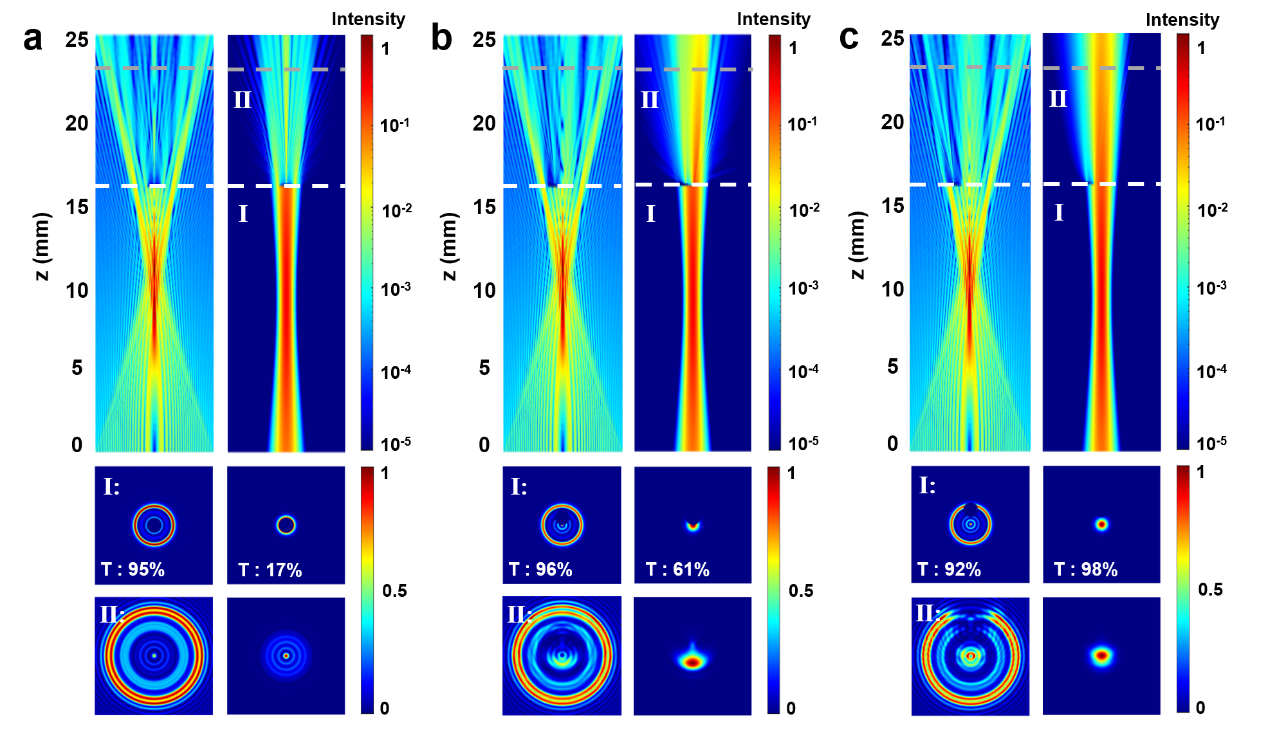


**Fig. S2** **Simulations of CAFAB and Gaussian beam intensity distribution at different occlusion. a-c** The x-z sections in log scale and x-y cross-sections in linear scale (I: white dash, II: gray dash) of Airy (left) and Gaussian (right) beams 3-D intensity profile when obstacle is positioned at (a) center of the beam, (b) 50 μm off-center, (c) 100 μm off-center.

For the feasibility of the adaptive UWOC system with the metasurface, we conducted simulations to investigate the performance of CAFAB and Gaussian beams at different occlusion. During the propagation of the beams, obstacles with a radius of 50-μm were introduced at position of *z* = 15.9 mm (white dash) in Fig. S2, and the beam intensity was calculated accordingly. Fig. S2(a)-(c) display the x-z sections of the beam intensity distribution when the obstacle is positioned at the center of the beam, 50 μm off-center, and 100 μm off-center, respectively. The left and right sections represent the beam intensities of Airy and Gaussian beams, respectively. The results demonstrate that Airy beams can gradually restore their ring structure during propagation when faced with obstacles at different positions, whereas Gaussian beams exhibit significant displacement and Poisson stripes due to diffraction. Illustrations I and II below correspond to the x-y cross-sectional diagrams of the beam intensity at the positions indicated by white and gray dashes in the figure. When considering obstacles of the same size at the three different positions, the transmittance of Airy beams is measured at 94.60%, 95.84%, and 92.25%, while the transmittance of Gaussian beams is significantly lower at 17.00%, 60.50%, and 98.00%. The results indicate that CAFAB exhibits superior robustness of transmittance compared to Gaussian beams.


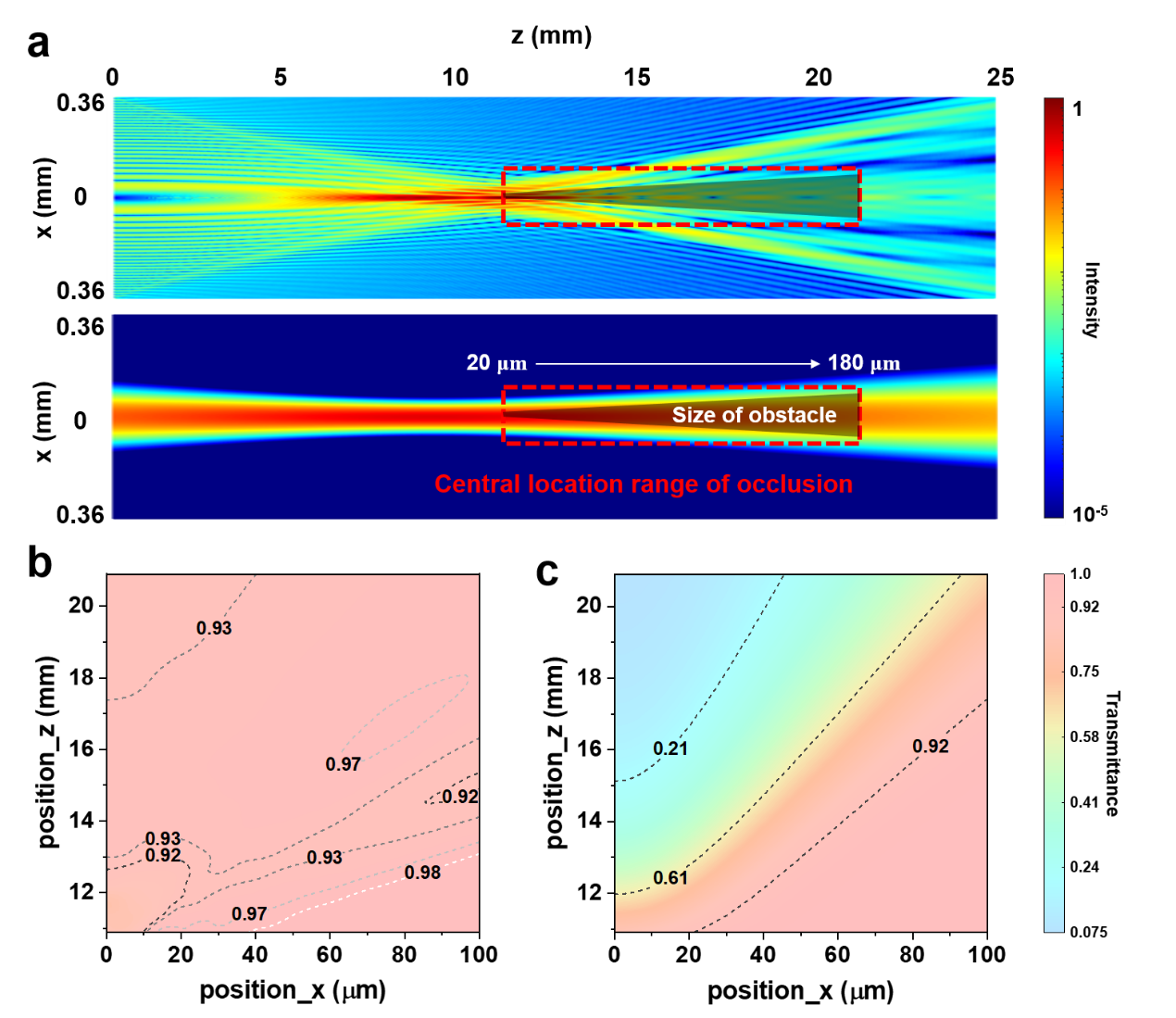


**Fig. S3. Simulation of CAFAB and Gaussian beam obstacle avoidance characteristics.** **a** The x-z sections of CAFAB (top) and Gaussian beam (bottom) propagation filed, The red dotted frame indicates the movement range of the center position of the circular obstacle, the black shadow indicates that the obstacle moves from 10.9 mm to 20.9 mm in +z direction, and its diameter increases from 20 μm to 180 μm accordingly. **b-c** Heat map of optical power transmittance versus obstacle position (b) CAFAB, (c) Gaussian beam.

We evaluate the anti-occlusion performance of CAFAB and Gaussian beam by simulate the changes in light power transmittance caused by obstacles of different sizes at different positions, which shown in Fig. S3. Gaussian beam waist radius is set to 30 μm, which equals to ring radius *r*_0_ of the Airy beam. The center movement range of the circular obstacle is -100 μm to 100 μm on the x-axis, 10.9 mm to 20.9 mm on the z-axis, and the diameter of the obstacle increases from 20 μm to 180 μm. Results in Fig. S3 (b) and (c) demonstrate Airy beam has stronger transmittance robustness than Gaussian beams when facing different occlusions. The average transmittance of Airy beam and Gaussian beam is 94.66% and 60.40%. And the standard deviation of the transmittance is 0.024 (Airy) and 0.31 (Gaussian), which proves that Airy beam has more stable obstacle avoidance characteristics.

**4. Experimental setup of RGB Airy beams based UWOC systems**


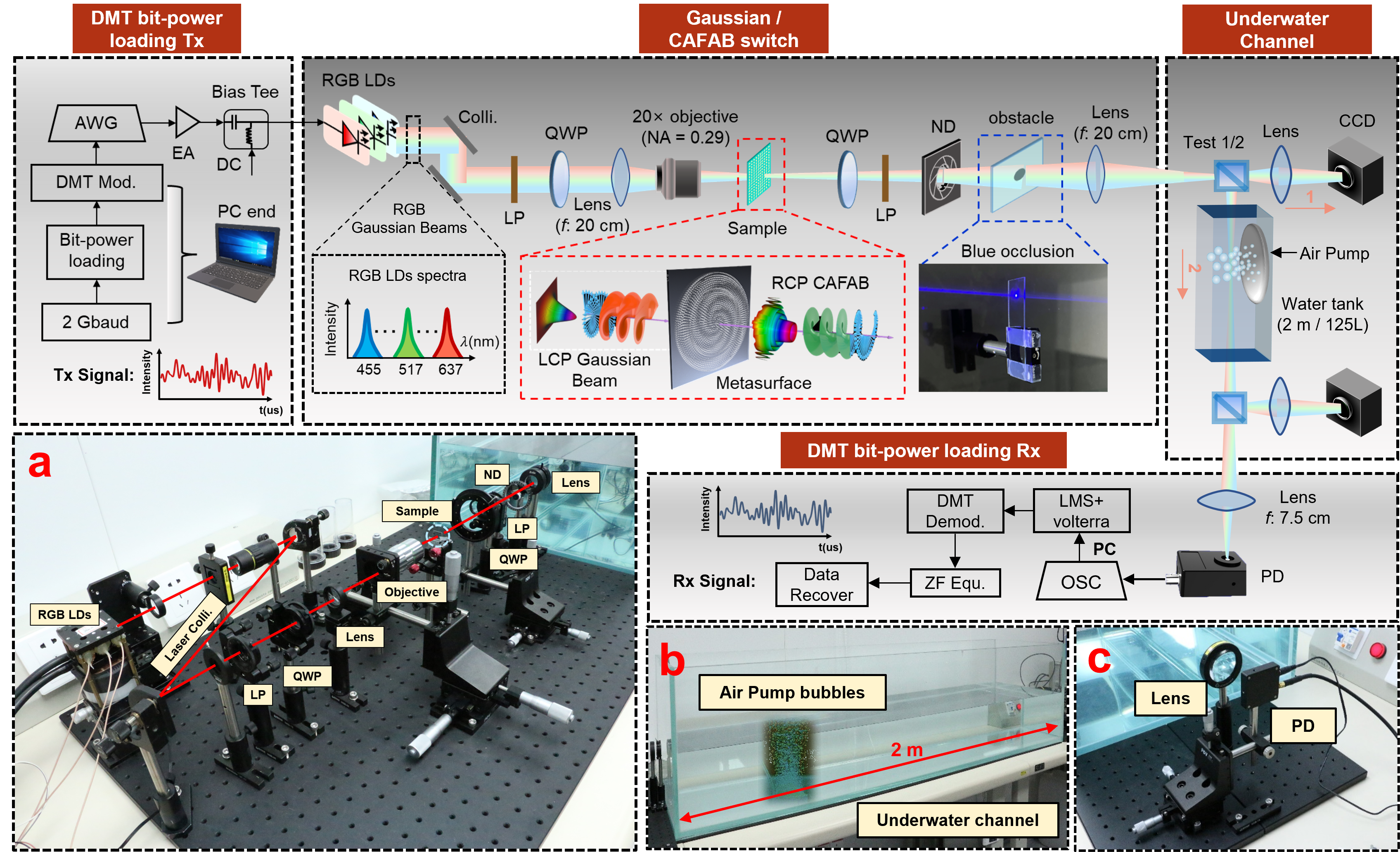


**Fig. S4. Experimental setup of the adaptive UWOC systems based on CAFAB metasurface for obstacle and bubble disturbance test.** **a-c** Photographs of the experimental setup (a) Transmitter optical path, (b) A 2-m underwater channel, (c) Receiver.

Fig. S4 shows the obstacle and bubble disturbance test setup of the CAFAB metasurface based UWOC system. Fig. S4 a-c show the photographs of Tx, underwater channel and Rx of our system. In the DSP at the transmitter, we generate a 2 Gbaud DMT bit-power loading modulated digital signal at the PC end. Here, we estimate the channel signal-to-noise ratio (SNR) by returning the measured error vector magnitude (EVM) from the quadrature phase shift keying (QPSK) signal. The SNR-Bits table can guide us to use Levin-Campello (LC) algorithm^2^ to allocate bits and power to each subcarrier of the channel, so as to maximize the channel spectral efficiency (SE). The modulated digital signal is directly adjusted on the tricolor lasers through a Bias-T after D/A conversion and electrical amplification. The tricolor spectra show the central wavelengths are at 455 nm, 517 nm and 637 nm^3^. For clarity, the tricolor channels are tested separately. The collimation system consists of two small holes and two mirrors, then the emission light from the laser diode is spatially filtered into a Gaussian beam, and then converted into a circularly polarized beam through a liner polarizer and an achromatic quarter-wave plate (QWP). The beam size is reduced by a 4f system consisting of a lens (*f* = 20 cm) and an objective (20×, NA = 0.29) to match the size of the metasurface. After the beam passes through the metasurface, Quarter-wave plate and polarizer ensure that the detected beam is cross-polarized. In fact, left-handed circularly polarized (LCP) Gaussian beam is transformed into right-handed circularly polarized (RCP) CAFAB after passing through the metasurface. An absorptive neutral density (ND) filter is used to prevents reception saturation and simulate attenuation with distance variation. In order to prevent unnecessary pollution of the water environment, obstacle consist of transparent glass coated with fixed-size black dye on both sides are fixed in place before entering the water. And the obstacle size is normalized to the beam aperture (*ω*_0_ = 14 mm). Then, communication and beam quality detection are measured separately. The intensity distribution is recorded by a CCD camera (MER-630-60U3M-L). During underwater transmission, we use an adjustable variable-speed air pump fixed at 1/4 of the water tank to simulate the influence of underwater bubbles, and adjusted the pump to 1 L/min, 6 L/min and 10 L/min respectively to generate small, medium and large bubbles. After being disturbed by underwater bubbles and attenuated by the environment, the beam out of the water is detected by a CCD or collected into a PD by a Lens. The received signal and the corresponding spectrum are displayed on the OSC. DSP at the receiving end mainly includes waveform-level LMS+Volterra post-equalization (taps of LMS and Volterra are 51 and 41), DMT demodulation and zero-forcing (ZF) equalization. The SE, data rate and BER can be calculated according to the recovered signal.

**5. Performance of RGB CAFAB based UWOC system in challenging environment**


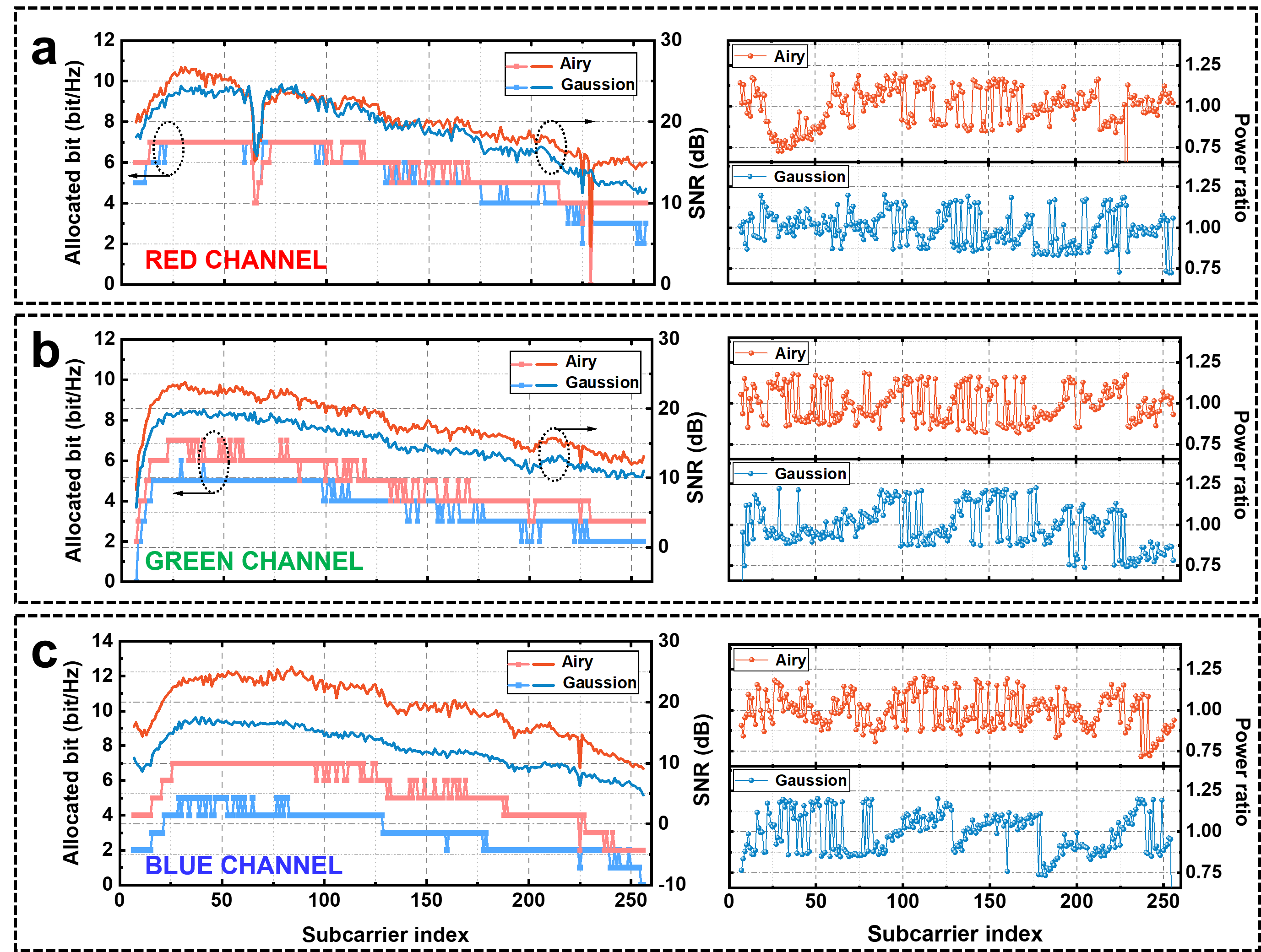


**Fig. S5. RGB channels bit-power allocation schemes at 0.43 *ω*_0_ occlusion.** **a** Red channel, **b** Green channel, **c** Blue channel.

Fig. S5 illustrates the bit-power allocation strategies for RGB Gaussian and Airy channels under 0.43 *ω*_0_ occlusion. The results reveal a significantly higher SNR for the RGB Airy channels compared to the Gaussian channels. The average subcarrier SNRs for the RGB Airy channels are 20.39 dB, 18.20 dB, and 19.19 dB, respectively, which are 1.20 dB, 3.11 dB, and 6.72 dB higher than those of the RGB Gaussian channels. The advantage of the Airy beam is most pronounced in the blue channel, while the performance of the Gaussian and Airy beams in the red channel is similar. Furthermore, the spectral efficiencies (SE) of the RGB Airy channels are 5.73 bits/Hz/s, 4.89 bits/Hz/s, and 5.34 bits/Hz/s, whereas the SEs of the RGB Gaussian channels are 5.32 bits/Hz/s, 3.80 bits/Hz/s, and 3.11 bits/Hz/s. The right portion of the figure depicts the power distribution results, where the fluctuation of the normalized power ratio on the initial allocation power of 1 corresponds to the upper and lower allocation of bits.

The specific values of ROP improvement are summarized in Table S1. The symbol ‘o’ in the table means that only Airy beam is supported at the effective data rate transmission, and the symbol ‘-’ means that neither Airy beam nor Gaussian beam can meet the threshold ROP (*P_th_*). Measured *P_th_* of RGB channels are -20.21 dBm, -17.52 dBm and -13.14 dBm.

**Table S1.** Improvement of ROP using CAFAB metasurface compared to RGB Gaussian channels versus size of obstacle.

| Size of obstacle (*ω*_0_) | Improvement of ROP using CAFAB metasurface (dB) | | |
| --- | --- | --- | --- |
|  | Red Channel | Green Channel | Blue Channel |
| 0 | + 0.02 | + 0.02 | + 0.01 |
| 0.07 | + 0.23 | + 0.32 | + 0.33 |
| 0.14 | + 0.23 | + 0.96 | + 0.46 |
| 0.21 | + 0.28 | + 1.27 | + 0.70 |
| 0.29 | + 0.82 | + 3.25 | + 1.99 |
| 0.36 | + 1.55 | + 4.24 | + 3.78 |
| 0.43 | + 2.41 | + 4.07 | + 7.85 |
| 0.57 | +3.40 | o | o |
| 0.71 | o | o | o |
| 0.86 | o | - | - |
| 1 | - | - | - |


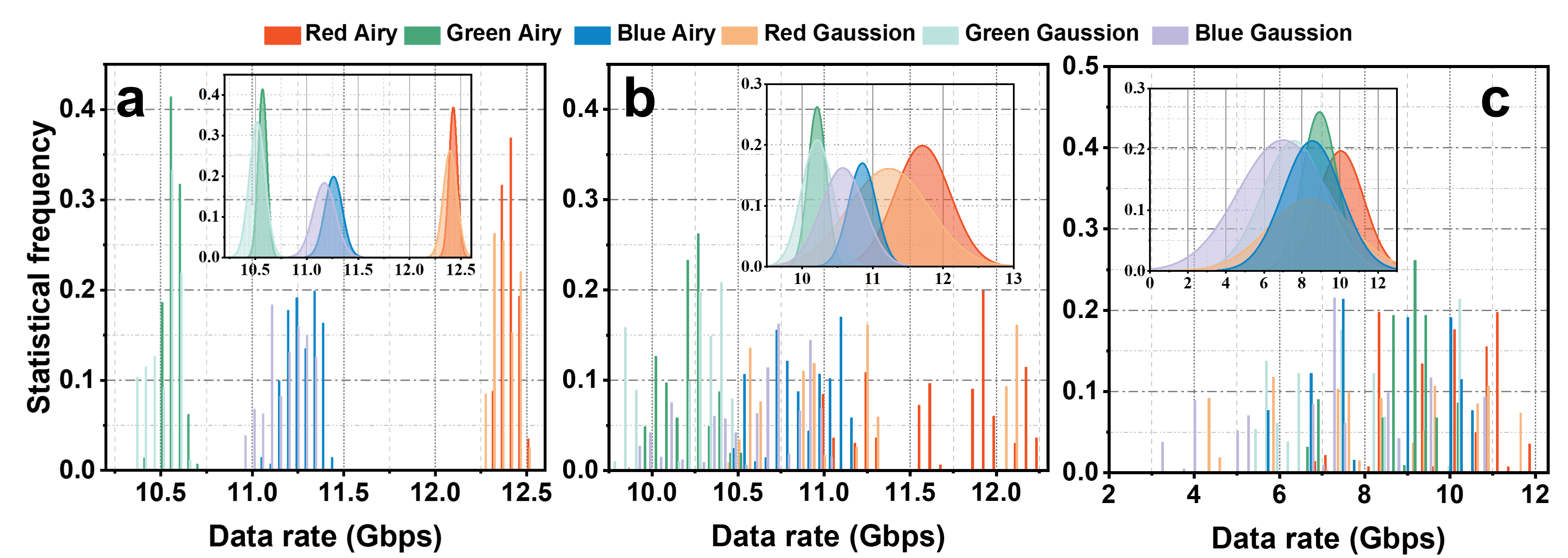


**Fig. S6. Transmission data rate jitter distributions of RGB Airy and Gaussian channels at air pump rate of** (**a**) 1 L/min, (**b**) 6 L/min, (**c**) 10 L/min.

To capture the oscilloscope spectrum jitter caused by air bubbles, we reduce the amount of transmitted data by half while increasing the scale of OSC time-base. The OSC sampling rate is set to 10 Gsa/s. For jitter outside the sync peak of a single sample, multiple samples are used to compensate. The obtained data rate distribution results are shown in Fig. S6. The insets in each panel represent the results of Gaussian fitting of probability distributions to the original data. The reason for the fitting choice is the jitter of the ROP presents a Gaussian-like distribution, while the rate of bit-power loading depends on the SNR related to the ROP level. Results indicate that RGB Airy beams exhibit superior stability under varying bubble conditions compared to Gaussian beams. At a pump rate of 1 L/min, the average data rate of the RGB Airy beams surpasses that of Gaussian beams by 0.03 Gbps, 0.05 Gbps, and 0.09 Gbps, while the standard deviations of the RGB channels decrease by 0.026 Gbps, 0.035 Gbps, and 0.024 Gbps, respectively. As the pump rate increases, the advantages of Airy beams become more pronounced. When the pump rate reaches 10 L/min, the average data rates of the RGB channels experience increments of 1.61 Gbps, 1.35 Gbps, and 1.53 Gbps, accompanied by reduced standard deviations of 0.98 Gbps, 0.75 Gbps, and 0.83 Gbps, respectively. The specific values are summarized in Table S2.

**Table S2.** Reduction in standard deviation of ROP and data rate with CAFAB metasurface compared to RGB Gaussian channels versus different bubble conditions.

| Air pump rate (L/min) | reduction in standard deviation with CAFAB metasurface | | | | | |
| --- | --- | --- | --- | --- | --- | --- |
|  | ROP (dB) | | | Data rate (Gbps) | | |
|  | R | G | B | R | G | B |
| 1 | 0.028 | 0.028 | 0.071 | 0.026 | 0.035 | 0.024 |
| 6 | 0.031 | 0.082 | 0.19 | 0.18 | 0.09 | 0.11 |
| 10 | 0.37 | 0.16 | 0.44 | 0.98 | 0.75 | 0.83 |


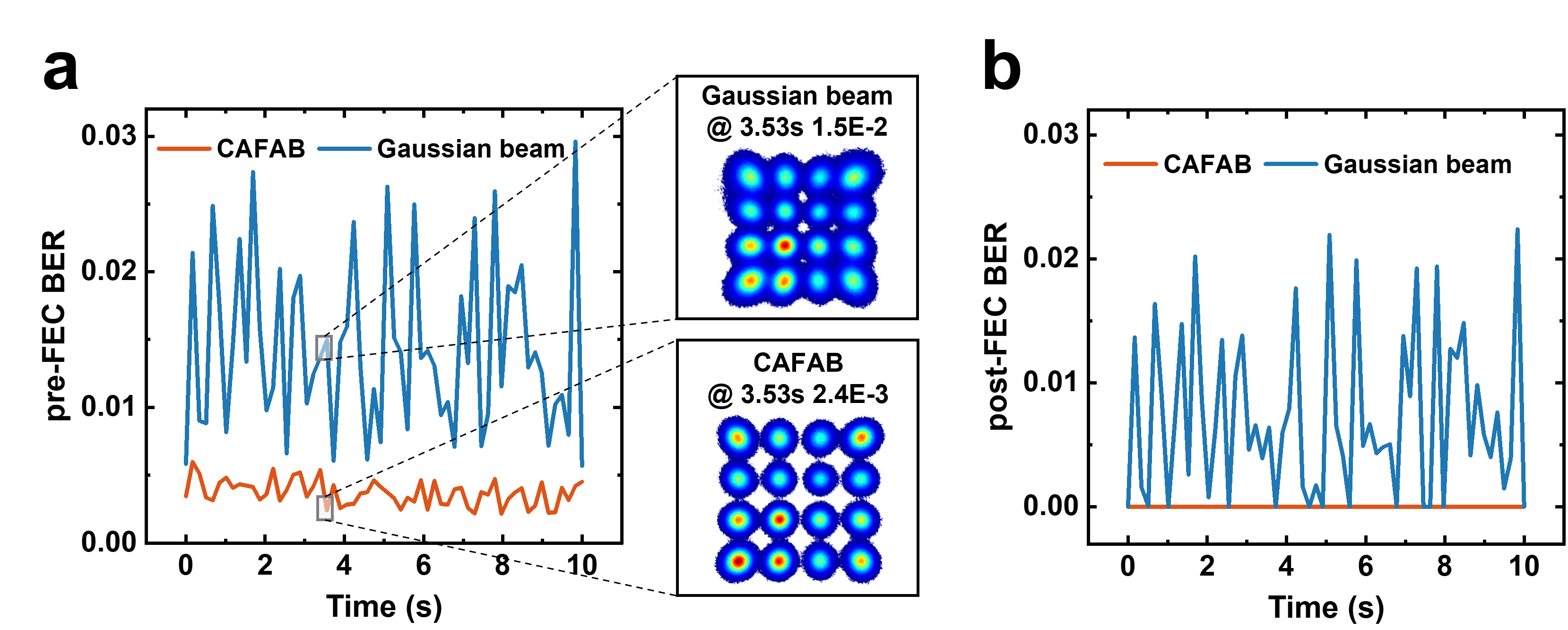


**Fig. S7. BER fluctuates over time in UWOC systems based on CAFAB or Gaussian beams:** (**a**) pre-FEC, and (**b**) post-FEC.

A BER analysis of the received video streaming is conducted, including pre-FEC and post-FEC BER. The results are displayed in Fig. S7. Fig. S7(a) shows the statistical results of pre-FEC BER. The BERs of the Gaussian beam-based system fluctuate dramatically over time, ranging from 5.7×10^-3^ to 2.9×10^-2^ with an average of 1.4×10^-2^. The pre-FEC BER of the CAFAB based system is more stable, averaging at 3.7×10^-3^. These results are also consistent with the statistical outcomes for data rate and ROP presented in Fig. 4 and Fig. 5 of the main text. When using the 10% low-density parity-check (LDPC) code as FEC encoding, all errors generated by the CAFAB based system can be corrected, resulting in no errors at the receiver, shown in Fig. S7(b). However, the Gaussian beam-based system can only recover the signal without errors at certain moments, which is already reflected in Fig. 6(c).


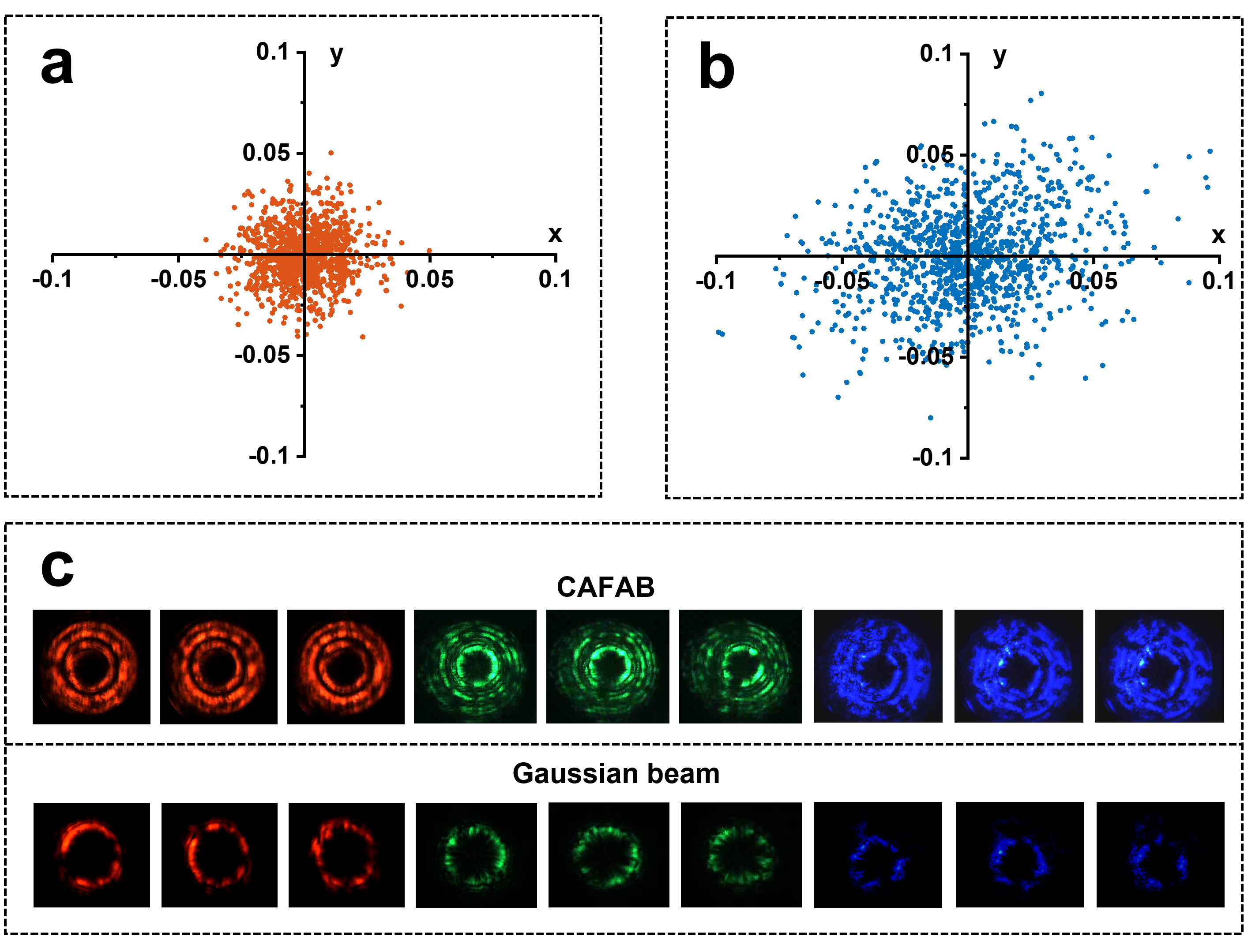


**Fig. S8. The beam quality of the received CAFAB and Gaussian beam in complex underwater channel.** Scatter plot of centroid position changes in beam clusters of (**a**) CAFAB, (**b**) Gaussian beams. **c** Selected CCD images of received CAFAB and Gaussian beams.

The comparison of the beam quality between received RGB CAFAB and Gaussian beam in 4K video communication is shown in Fig. S8. In terms of the variations in the centroid positions of the received beam clusters, the CAFAB clusters exhibit a more stable intensity distribution, with centroid position drift ranging only between -0.05 and 0.05 on the normalized x-y axes and an average offset of 1.6×10^-2^, as shown in Fig. S8(a). For Gaussian beam clusters, the range of centroid movement extends to between -0.1 and 0.1 on the biaxial scale, with twice the offset of CAFAB, 3.0×10^-2^, as shown in Fig. S8(b). These results also directly demonstrate the superior robustness of the CAFAB based UWOC system when facing underwater bubble interference. Selected received RGB beam CCD images are displayed in Fig. S8(c), showing that CAFAB reshaped by the full-color metasurface maintains higher and more stable ROP when faced with obstructions and bubbles. The average ROP of the RGB channels in the CAFAB based system, compared to the Gaussian beam-based system, has increased by 2.57dB, 3.48 dB, and 7.31dB respectively. This provides significant assurance for 20 Gbps 4K video transmission in the complex underwater environment.

**References**

1. Minghelli-Roman, A., Goreac, A., Mathieu, S., Spigai, M. & Gouton, P. Comparison of bathymetric estimation using different satellite images in coastal sea waters. *Int J Remote Sens*. **30**, 5737-5750 (2009).
2. Campello, J. Practical bit loading for DMT. In *Proc IEEE International Conference on Communications (ICC)*, Vancouver, BC, Canada, pp. 801–805 (IEEE, 1999). https://doi.org/10.1109/ICC.1999.765384.
3. Hu, J. et al. 46.4 Gbps visible light communication system utilizing a compact tricolor laser transmitter. *Opt. Express*. **30**, 4365-4373 (2022).
